# Technology Anxiety on Attitude Towards Technology: The Moderating Effect of Age-friendly Environment

**DOI:** 10.1093/geroni/igab046.2490

**Published:** 2021-12-17

**Authors:** Do Kyung Yoon, Seolah Lee, DaeEun Kim, Chang Oh Kim, Hey Jung Jun

**Affiliations:** 1 Yonsei University, Seoul, Seoul-t'ukpyolsi, Republic of Korea; 2 Severance Hospital, Yonsei University College of Medicine, Seoul, Seoul-t'ukpyolsi, Republic of Korea

## Abstract

The purpose of this study was to examine the moderating effect of an age-friendly environment on the relationship between technology anxiety and attitude towards technology among Korean older adults. We collected data by online surveys in February 2021, and the sample was 324 Korean older adults aged 65 and above. The dependent variable was the attitude towards technology, which meant the appraisal about using a wearable robot for exercise. The independent variable was technology anxiety, meaning an individual’s apprehension of using a wearable robot. The moderating variable was age-friendly environment, which comprises domains of the physical environment, social environment, and municipal services. The higher the score is, the more age-friendly the environment was perceived. Control variables were age, sex, education, household income. The moderation effect was estimated by bootstrapping and PROCESS macro. Results showed that when older adults showed a higher level of technology anxiety, their attitude towards technology was less positive. Moreover, the moderation effect of an age-friendly environment was significant. Concretely, in the case of living in a less age-friendly environment, older adults with a higher level of technology anxiety were more likely to report a less positive attitude towards technology. However, the effect of technology anxiety on attitude towards technology was not significant among older adults living in a more age-friendly environment. It suggested that a practical intervention to reduce the level of technology anxiety is in need in order to promote a positive attitude towards technology, especially for older adults living in a less age-friendly environment.

